# Multimodal Assessment of Changes in Physiological Indicators when Presenting a Video Fragment on Screen (2D) versus a VR (3D) Environment

**DOI:** 10.1155/2022/5346128

**Published:** 2022-11-28

**Authors:** Elena Kriklenko, Anastasia Kovaleva, Aleksei Klimenko, Usman Dukuev, Sergey Pertsov

**Affiliations:** P.K. Anokhin Research Institute of Normal Physiology, Moscow, Russia

## Abstract

The increasing role of virtual environments in society, especially in the context of the pandemic and evolving metaverse technologies, requires a closer study of the physiological state of humans using virtual reality (VR) for entertainment, work, or learning. Despite the fact that many physiological reactions to the content presented in various modalities under VR conditions have already been described, often these studies do not reflect the full range of changes in the physiological reactions that occur to a person during their immersion in the virtual world. This study was designed to find and compare the most sensitive physiological indicators that change when viewing an emotionally intense video fragment in standard format on screen and in virtual reality conditions (in a VR helmet). The research methodology involved randomly presenting a group of subjects with visual content—a short video clip—first on screen (2D) and then in a virtual reality helmet (3D). A special feature of this study is the use of multimodal physiological state assessment throughout the content presentation, in conjunction with psychological testing of the study participants before and after the start of the study. It has been discovered that the most informative physiological indicators reflecting the subjects' condition under virtual reality conditions were changes in theta rhythm amplitude, skin conductance, standard deviation of normal RR-intervals (SDRR), and changes in photoplethysmogram (PPG). The study results suggest that in the process of immersion in a virtual environment, the participants develop a complex functional state, different from the state when watching on screen, which is characterised by the restructuring of autonomic regulation and activation of emotion structures of the brain.

## 1. Introduction

Currently, more than two hundred engineering companies around the world are making and researching virtual reality (VR) equipment. VR's entertainment potential is beyond question [1]. However, in addition to entertainment, there are other applications for this technology. For example, VR is quite widely used for the rehabilitation of patients with musculoskeletal pathologies [2, 3], Parkinson's disease [4, 5], or for acute brain circulation disorder [4, 6]. It has also been demonstrated that being in VR is effective to normalise autonomic indicators in severe stress [7].

In addition, VR systems are used in learning processes to increase student productivity and motivation [8]. Virtual environments provide a distinct effect of presence, engagement, and empathy, making viewing educational content more emotional [9]. In particular, there is evidence of the positive effects of virtual reality on training physicians' surgical skills: multitasking, ability to precisely perform manipulation, and better coordination [10–12].

However, despite the subjective reports showing that most subjects report a stronger engagement when perceiving information or experiencing emotions in the virtual environment, objective indicators of the autonomic nervous system and brain activity in VR are more ambiguous.

## 2. Related Works

The literature describes the results of a large number of works indicating how virtual environment influences the change in psychophysiological parameters and the emotional state of a person, but the data presented is largely contradictory. Thus, in some studies, only the subjective assessments of the subjects were taken into account; in these cases, participants described viewing emotional video clips in VR as more exciting than viewing content on a usual screen [13–18]. Other studies do not show any significant differences in the strength of the emotional response between viewing a scenario on screen and in a virtual reality helmet [19].

In most cases, objectification of emotional reactions is performed through the indicators of the cardiovascular system, skin conductivity, muscle tension, respiratory parameters, and bioelectrical activity of the brain.

### 2.1. Cardiovascular and Autonomic System Performance

It has been shown in some studies that unpleasant scenes cause a relatively much slower heart rate than pleasant ones when viewing usual pictures, suggesting the influence of the emotion type on the heart rate [20], and that the heart rate when watching a movie in a virtual environment is greater than at rest [7]. However, the heart rate is influenced by a large number of factors, so heart rate variability indicators are considered to be the more correct indicators. In particular, respiratory sinus arrhythmia can be used to assess levels of activation and affective responses [21].

In their studies, Nardelli et al. [22] and Valenza et al. [23] applied Russell's axial model [24] and presented stimuli from the International Affective Digital Sounds (IADS) base [25].

Heart rate variability indicators were used as a predictor of emotion. The following indicators changed significantly: mean RR-interval duration, standard deviation of RR-interval duration, RMSSD, TINN, LF%, LFnu, HF%, HFnu, LF/HF, and nonlinear HRV analysis indicators—SD1, SD2, SD12, and S (Poincaré plot). The precision of emotion detection was 84.72% for valence and 84.26% for power of emotion.

When analysing heart rate variability indicators, it was detected that intense stimulation under virtual reality conditions leads to an increase in HF power and a decrease in LF/HF ratio [26].

### 2.2. Electrodermal Activity

Skin conductance (or the inverse value and skin resistance) is one of the most sensitive indicators of unconscious processes. Skin conductivity indicator is often used in research devoted to emotion because it varies significantly with the level of activation evoked by emotional stimuli [27].

When studying the reactions to images assessed on a two-dimensional arousal-valence scale, it has been shown that autonomic indicators, including skin conductivity, are related to subjective evaluations of the emotionality of visual stimuli [20]. Early work showed that skin conductivity increases monotonically with increase in stimulation intensity [20]. Besides, in most cases, it turns out that skin conductivity is directly related to the level of activation but does not depend on whether the stimulus is pleasant or unpleasant; i.e., it does not depend on the sign (valence) of the emotion [28]. There is evidence that the level of skin conductivity did not differ significantly when the stimuli were presented in a virtual environment. But according to some data, the level of skin electrical activity increases when using VR [15].

### 2.3. Breathing

Breathing, which is an autonomic function, is also closely related to emotion. A study by [29] showed that there are cortical projections to stem respiratory neurons. This means that behavioural influences originating in higher brain centres can modify breathing patterns. The relationship between emotion and breathing is reflected in faster breathing in the state of activation [30]. The relationship between emotions and respiratory responses to natural noises and unpleasant sounds has also been investigated [31–33]. All studies note that when you change the emotional state, respiratory rate varies quite significantly.

### 2.4. Brain Activity

In most studies, where one of the study objects was the electroencephalogram (EEG), it has been demonstrated that the total EEG power decreased under conditions of psychoemotional stress [13, 14]. However, other studies indicate a greater power of the EEG beta rhythm while being in virtual reality [13, 14, 19]. The information about the character of alpha rhythm power changes under VR conditions is contradictory. Thus, according to Fadeev et al. and Tian et al., this index increased when viewing emotionally significant content, which may be related to the activity of sensory inhibition mechanisms in the most stressful situations [13, 18]. They have shown that positive emotions evoke significantly more alpha activity than negative and neutral emotions when shown videos of different emotional content under VR conditions. When studying various physiological measures in VR, Marín-Morales et al. [26, 34, 35] found that the most pronounced changes in characteristics were related to EEG rather than HR.

However, according to some data, no significant changes in EEG alpha rhythm power were obtained [14, 36]. In addition, there is contradictory information about EEG specifics during content presentation in virtual reality; according to some data, there are no significant differences in EEG frequency power data between real and virtual conditions [36], while other data show that the total EEG power in VR decreased [14].

Thus, a review of a number of articles comparing changes in physiological indicators when presenting the stimuli in the usual mode on screen and in virtual reality showed that currently there is no unequivocal idea of the direction of changes in both autonomic functions and encephalogram parameters.

In addition to the data, indicating multidirectional changes in physiological indicators during the presentation of emotional stimuli in the VR environment, the analysis of the literature showed that there are certain difficulties in comparing the results of similar studies. This is due to the fact that the study groups use stimuli of different modality (static images or video fragments), the time of content presentation can vary from several seconds to an hour, and the equipment is drastically different. In addition, the effect of the equipment itself on the test subject's condition is often not taken into account. For example, studies assessing EEG power when using a VR helmet note a significant increase in the power of high-frequency rhythms [37], which is most likely an artefact of the helmet attachment, but this result is discussed in the context of the brain bioelectrical activity, which is most likely incorrect.

Thus, despite a growing number of studies on the immediate reactions and objective signs of human engagement when presented with various kinds of stimuli in virtual reality, there is still much contradictory data on the dynamics of physiological processes under different conditions of presentation of emotionally saturated content.

This study was aimed at finding and comparing the most sensitive physiological indicators that change when viewing an emotionally intense video fragment in usual mode on screen and in virtual reality conditions (in a VR helmet).

## 3. Materials and Methods

The procedure followed the general criterion of the local ethics committee, based on the Helsinki Declaration principles, and was approved by the Bioethics Committee of the P.K. Anokhin Research Institute of Normal Physiology, Moscow.

### 3.1. Participants

Eighteen young men aged 19-23 years participated in the study (mean age 20.53 ± 1.23 years and median age 21 years). All participants were somatically healthy, denied bad habits, and had no neurological problems at the time of the study. All participants were informed in advance about the goals and objectives of the research project, as well as about the absence of health risks during the study, and signed a voluntary informed consent to participate in the study.

In order to avoid the occurrence of the virtual reality disease, we chose a short fragment for the presentation.

Nonetheless, we asked our participants to self-report whether they felt any negative symptoms or not while watching the video in the VR environment. None of them noted any significant signs of fatigue or discomfort.

Eleven subjects (61%) had visual acuity problems—myopia (-0.5 to -5.5)—and one subject had astigmatism, which had been corrected at the time of study participation. There were no problems with stereo vision or colour perception in the subjects.

### 3.2. Instruments and Measures

The comparison groups in our study consisted of the same participants who viewed the same content randomly on screen and in a virtual reality helmet. Participants were first introduced to the content of the video segment through screenshots to avoid the novelty effect.

The study included several stages. Before the main stage of the study, all participants were asked to fill out the following psychological tests: the Spielberger-Hanin State-Trait Anxiety Inventory (STAI) [38] and the Toronto Alexithymia Scale (TAS, Russian adaptation) [39].

In the main phase of the study, subjects were presented with a video clip that contained emotionally relevant content.

We used a 360 video fragment adapted for virtual reality environment. The video clip was emotionally significant (a short horror movie fragment) and was obtained from open Internet sources. The participants were in a sitting position in front of the monitor throughout the study. VR helmet was worn by participants during the stage of 3D-mode content demonstration.

**Figure 1:**
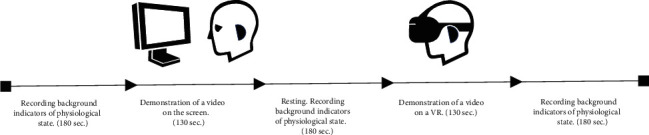
Scheme of the main stage of the study.

The video clip was shown on screen and in a virtual reality helmet in a randomised order (some participants watched the fragment on screen first, while others watched it in the VR helmet). The duration of the video segment was 130 seconds (Figure 1).

It is known that the sign of the experienced emotion has a greater influence on physiological indicators than the way the stimulus is presented [18], and negative stimuli elicit a greater reaction than positive ones (and, consequently, than neutral ones). In this regard, for the present study, a negative valence and a short duration video segment (130 seconds) was chosen in order to avoid the development of fatigue signs.

**Figure 2:**
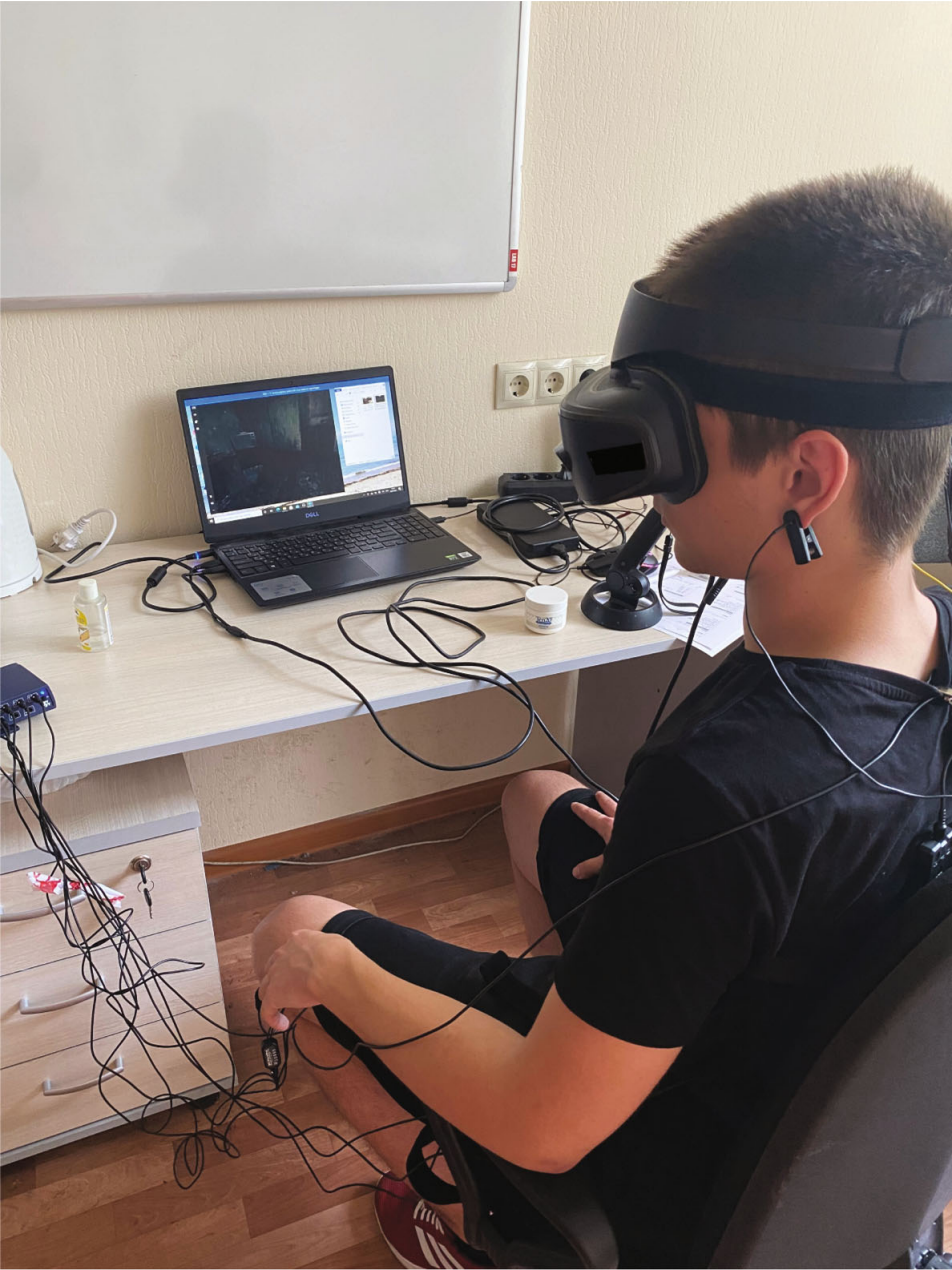
Experimental procedure.

To demonstrate the video, the Lenovo Explorer virtual reality helmet was used. It runs on the Windows Mixed Reality virtual environment, a mixed reality platform, presented as part of the Windows 10. A laptop Dell G5 5500 with the following parameters was used: core (ТМ) i7 10750Н, NVIDIA GeForce RTX 2070. The video file was demonstrated to the participant on a 15^″^ monitor at a distance of no more than 1 m (Figure 2).

Between watching the video in different modes, a 180-second rest was provided. While watching the clip, the subjects sat in a chair without having to move around the room or turn their head.

The following physiological indicators were recorded for 180 seconds before the main stage of the study, as well as throughout the main stage of the study: EEG (monopolar from Cz lead), photoplethysmogram (PPG) from left thumb, skin conductivity, electromyogram (EMG) of forehead muscles, respiratory frequency, and amplitude. Physiological indicators were recorded using a polygraph by Thought Technology (Canada). The duration of the entire study for one participant was 13 min.

**Figure 3:**
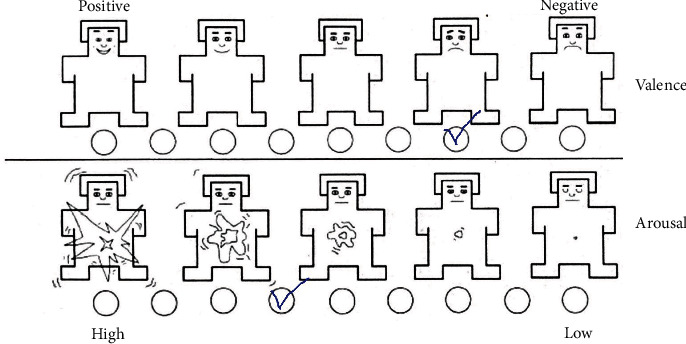
The Self-Assessment Manikin (SAM).

After the main phase of the study, participants completed the Self-Assessment Manikin [40] (Figure 3). Participants had to evaluate the watched fragment on two scales: the strength of the evoked emotion (arousal) and the sign of the emotional reaction (valence).

During the video, the action within the story was not evenly distributed. The first and last 30 seconds of the clip did not contain any events and were the background to assess the impact of the main fragment. Further, according to the clip plot, we identified the three most emotional episodes to which the participants showed the most pronounced reaction; the last part of the clip also did not contain any emotional moments.

### 3.3. Statistical Analysis

Data analysis was performed using appropriate polygraph software (BioGraph Infiniti) and standard statistical processing software packages.

Due to large individual differences in physiological parameters, it was decided to use normalised values for analysis.

Physiological indicators during viewing were assessed relative to the previous background state of the participants. Relative changes in the indicators were calculated compared to the resting state with eyes open (control) using the following formula:

(1) $$\begin{array}{c} X=\left(\text{Xn}÷\text{Xo}\right)\times 100\%, \end{array}$$

where Xn is the value of the current indicator and Xo is the value of the indicator in the baseline state for this subject.

An index value equal to one meant that there were no changes relative to the rest state.

Thanks to the normalised representation of the data, we were able to compare indicators with different units of measurement.

The nonparametric Mann–Whitney criterion was used for statistical analysis of the values of differences in physiological indicators when a video fragment was shown on screen and in a virtual reality helmet.

## 4. Results and Discussion

### 4.1. Results

According to the results of psychological testing, the participants of the study had an average or low level of alexithymia and an average or low level of anxiety (Table 1).

**Figure 4:**
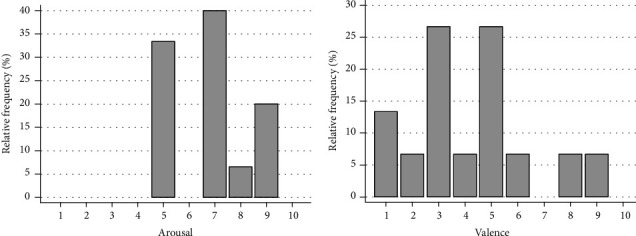
The Self-Assessment Manikin (SAM) result distribution in the sample.

The presented video fragment contained emotionally intense content—a fragment of a horror movie. The results of the self-assessment of the emotions evoked by this fragment are presented in Figure 4.

The analysis of subjective assessment showed that the presented video fragment provoked quite a strong emotional reaction in the majority of the participants (power of emotion 6.8 ± 1.52 and median value-7) and the evaluation of the sign of the emotion experienced was divided; despite the expected negative characteristic of the video fragment, many evaluated the feeling they experienced during viewing as positive (value of the emotion sign 4.2 ± 2.31 and median value-4).

#### 4.1.1. Changes in Cardiovascular Indicators

**Figure 5:**
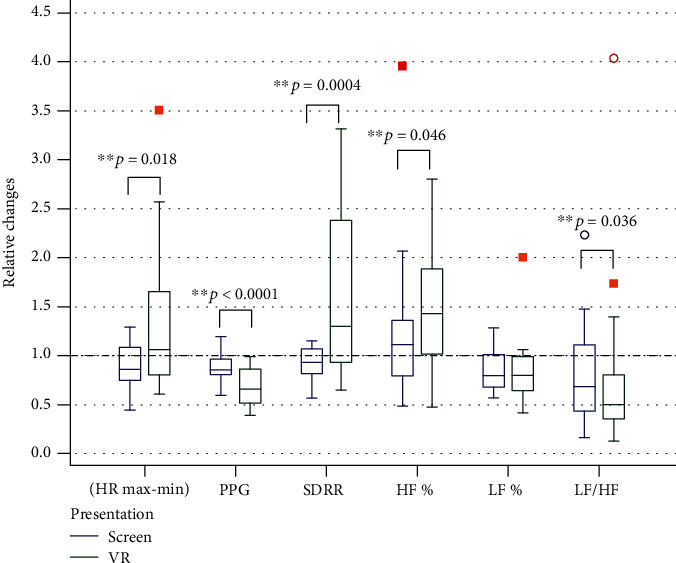
Relative (to prior background) changes of cardiovascular parameters during watching video in 2D and 3D mode (HR max-min: delta between maximum and minimum heart rate; PPG: photoplethysmogram amplitude; SDRR: standard deviation of RR-intervals; HF%: relative power of high-frequency waves in HRV spectrum; LF%: relative power of low-frequency waves in HRV spectrum; LF/HF: LF waves to HF waves ratio). “1” means background.

When analysing the indicators of cardiovascular system state (Figure 5), we calculated the following values: photoplethysmogram (PPG), average HR value, difference between maximum and minimum HR values, standard deviation of normal RR-intervals (SDRR), and indicators of spectral (frequency) analysis of heart rhythm variability (relative LF, HF values, and LF/HF ratio).

As you can see in Figure 5, the two interrelated indicators—HR max-min and SDRR—show opposite dynamics relative to the background when watching content on screen (2D) and in a virtual reality helmet (3D); in the first case, the value decreases relative to the baseline state, and in the second, it increases.

The heart rate increases (compared to the previous baseline state) while using virtual reality and does not change or even decreases while watching a video clip on screen. Decrease in photoplethysmogram (PPG) when viewed in a VR helmet is more pronounced than when the clip is presented on screen.

#### 4.1.2. Changes in Respiratory Parameters

**Figure 6:**
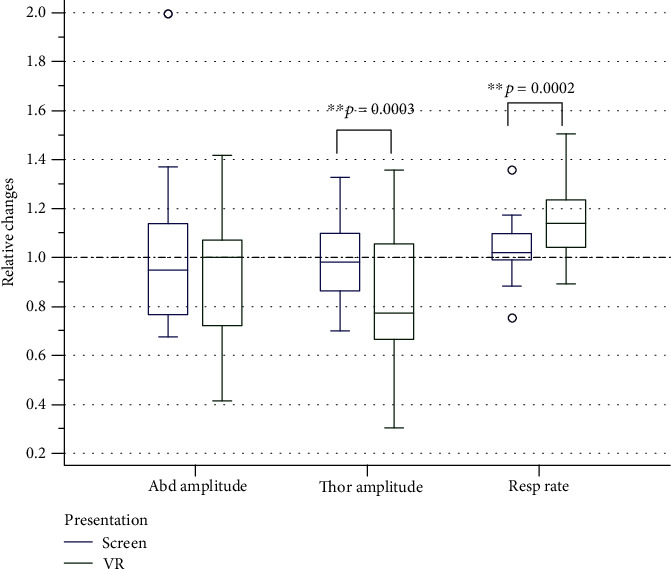
Relative (to prior background) changes of breathing parameters during watching video in 2D and 3D mode. “1” means background.

Both abdominal and thoracic breathing and respiratory rate were recorded in the study participants Figure 6.

When watching a video fragment in the VR helmet, the amplitude of thoracic breathing decreased and respiratory rate increased (compared to the previous baseline state). The participants, who viewed the video on screen (2D), did not have any changes in breathing parameters compared to the baseline state.

#### 4.1.3. Changes in EEG Readings

**Figure 7:**
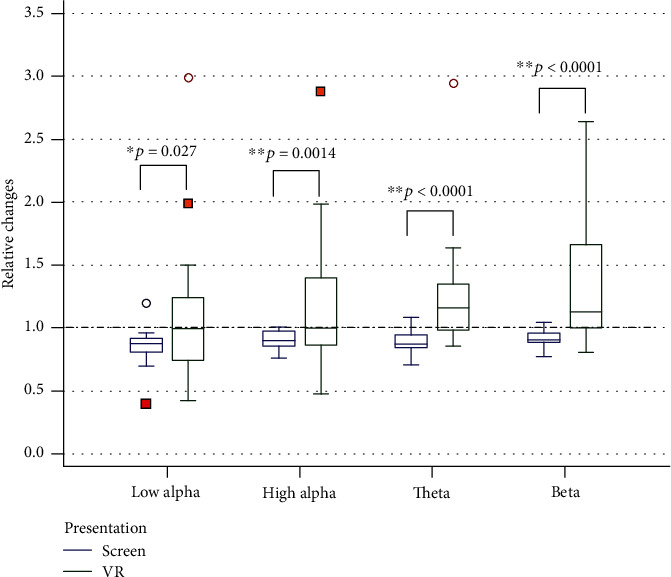
Relative (to prior background) changes of EEG parameters during watching video in 2D and 3D modes. “1” means background.

The equipment we used allowed us to record only one electroencephalogram channel. In our study, the electrode was placed on the Cz area, which helped to reduce artefacts from the virtual reality helmet attachment and obtain a signal containing all major rhythmic components: theta (4-7 Hz), alpha 1 (8-9.5 Hz), alpha 2 (10-13 Hz), and beta oscillations (14-35 Hz) of EEG (Figure 7).

Figure 7 shows that the spectral power of all EEG rhythms increases when watching a video in a VR helmet, which may be related to the appearance of physiological (related to muscle tension) or physical (due to the characteristics of the VR helmet) artefacts. The most prominent increases are in the beta and theta bands, which correlate strongly with the value of the electromyogram [37].

#### 4.1.4. Changes in Other Physiological Indicators

**Figure 8:**
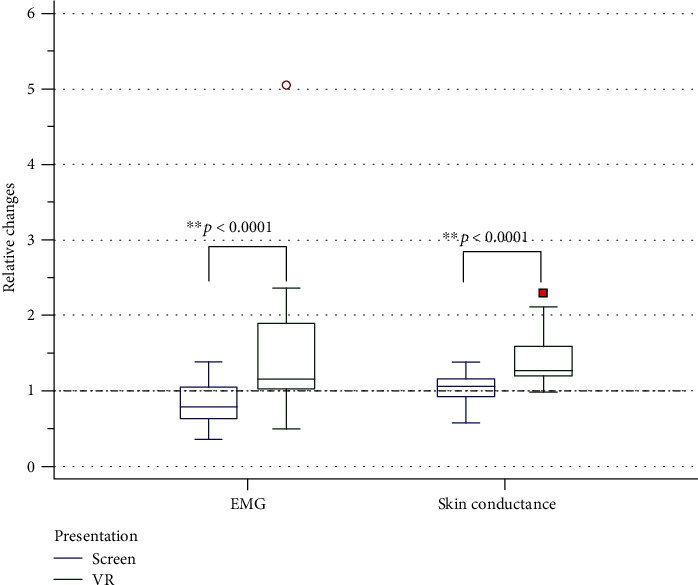
Relative (to prior background) changes of EMG and skin conductance parameters during watching video in 2D and 3D modes. “1” means background.

In addition to changes in cardiovascular system values, respiration and EMG, skin conductivity, and electromyography demonstrated significant differences in values when watching a video fragment in a VR helmet, which increased compared to the baseline state (Figure 8).

**Figure 9:**
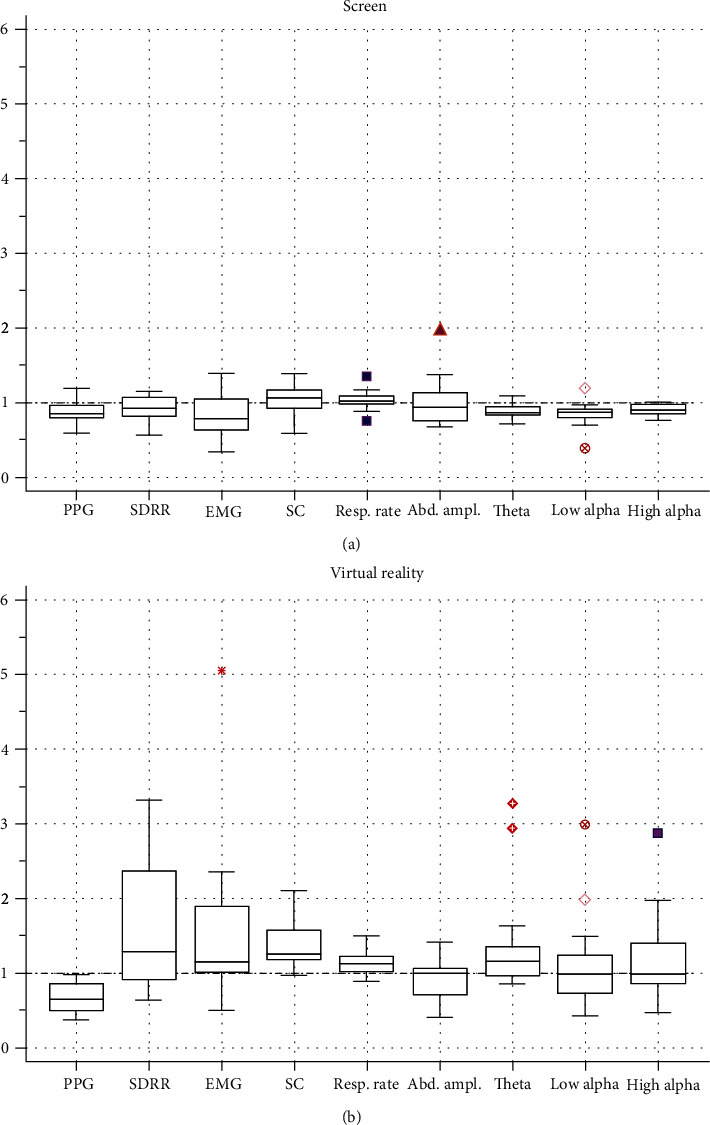
Changes in the most informative physiological indicators relative to the initial level when watching videos on the screen (a) and in virtual reality (b). PPG: photoplethysmogram amplitude; SDRR: standard deviation of RR-intervals; EMG: electromyogram; SC: skin conductance; resp. rate: respiratory rate; Abd. ampl.: abdominal breathing amplitude; theta: 4-7 Hz; low alpha: alpha 1 (8-9.5 Hz); high alpha: alpha 2 (10-13 Hz).

Thus, when demonstrating a video fragment in the virtual reality mode, we identified the most informative physiological indicators, which are those that change the most relative to the baseline state (Figures 9(a) and 9(b)).

We consider such “informative” physiological indicators as changes of theta rhythm amplitude, skin conductance, SDRR, and changes of photoplethysmogram amplitude (PPG). The values of these indicators change relative to the baseline state more than any other and show statistically significant differences when the video fragment is shown on screen and in the VR helmet.

## 5. Discussion

In the present study, we used randomised content presentation: half of the participants watched the fragment first on screen and then in the VR helmet, while the other half of the participants watched it in reverse order. In addition, participants were introduced to the content of the video beforehand (several screenshots were shown while electrodes were attached), which allowed us to compare reactions to the nature of the presentation in the same subjects and avoid novelty effects for both the presentation of content on screen and in the VR helmet.

As in the majority of studies investigating emotional reactions when using VR, the present study found that the content presented in VR caused greater emotional excitement in the subjective evaluation of the participants when compared to viewing it on screen.

The data obtained is generally consistent with similar studies [18]. Thus, according to Peperkorn et al. [41], showing “negative” clips in a virtual environment can increase emotional excitement when compared to showing them on screen.

Nevertheless, subjective feelings should not be the only indicators of human involvement and immersion in the offered content, as a number of works note that visual stimuli presented to participants in VR may not cause more emotional excitement in comparison with content demonstration in 2D mode. In this regard, the present study used multimodal registration of objective physiological indicators during video viewing both on screen and in the VR helmet.

In assessing cardiovascular performance, the present study demonstrated an increase in heart rate spectral analysis, such as HF%, when presented with a video clip in a VR helmet. Accordingly, we observed a decrease in the LF/HF ratio (with the LF% value unchanged), indicating the predominance of parasympathetic influences on heart rhythm regulation under the indicated conditions. Similar results have been demonstrated by many [34, 42]. In particular, in the study of Sokhadze [42], where the participants were presented with images of mutilated bodies, there was an increase in HF power and a decrease in the LF/HF ratio. Shenhav and Mendes [43] demonstrated short video clips of bodily injuries to the study participants and found that such content caused higher reactivity of the HF component.

Changes in EEG parameters in the participants of the present study were associated with changes in the spectral power of all EEG rhythms, which increased when watching the video in the VR helmet compared to watching it on screen. The most noticeable increase was specific to fluctuations in the beta and theta ranges. Similar data is presented by a number of authors [13, 14, 17]. The increase of alpha rhythm is shown in the study by Tian et al. [18], where it was also noted that positive affects cause a more significant increase in this index.

Regarding the growth of high-frequency activity (starting from the beta range), we should note its high correlation with the tension of the muscles of the face, head, and neck [38]. Given that, in our study, the power of the forehead EMG was also significantly higher when viewing the clip in virtual reality, there is a reason to believe that the increase in the high-frequency components of the EEG is a sign of higher muscle tension when the helmet is worn.

As for theta activity, which in our study also increased when a stimulus was presented in the VR helmet, it is known to play a key role in the operation of attention systems [44], spatial and temporal signal encoding, memory, in providing behaviour related to anxiety [45], and such states as interest [46]. Interest as a positive emotion is associated with the ability to absorb new information and/or better understand already generalised information, as well as increased attention, information processing, learning, and motivation [46]. Electrical activity in the theta band in the frontal leads increased compared to the rest state in the case of concern [46].

In the present study, we found that the skin conductance increased when the video fragment was presented to the subjects on screen and to an even greater extent when it was presented in the VR helmet, which indicates a more pronounced activation of the sympathoadrenal system under the described conditions. Despite the fact that skin electrical activity is traditionally considered to be a reliable indicator of the sympathetic nervous system's response to affective stimuli, some works have shown that skin conductivity under virtual reality conditions does not differ significantly from the baseline values [41]. Some studies have noted insignificant changes in this parameter [47].

Thus, we can assume that the aggregate results of the assessment of changes in physiological indicators and psychological tests when presented with video content in virtual reality suggest that the selected stimulus caused interest rather than fear or surprise in the participants of this study, and it is more pronounced when presented in the VR helmet compared to the screen.

## 6. Conclusions

Results of multimodal physiological assessment when watching video on screen and in a virtual reality helmet suggest greater involvement of participants in the VR environment.

Despite the chosen content (a fragment of a horror movie), it is more likely that participants felt interest rather than fear or consternation.

The most informative physiological indicators reflecting the subjects' condition under virtual reality conditions were the following: theta rhythm power, skin conductivity, standard deviation of RR-intervals (SDRR), and blood volume pulse amplitude.

Based on the obtained results, we can suggest that in the process of immersion in a virtual environment, the participants develop a complex functional state different from the state when watching on screen, which is characterised by the restructuring of autonomic regulation and activation of emotional structures of the brain.

## 7. Limitations

The results presented in this study have to be interpreted in the light of some limitations. We used male participants only because it is known that females during different stages of menstrual cycle demonstrate significant changes in the physiological parameters that we used to assess the condition of the participants: heart rate variability [48] and EEG parameters [49]. So it is necessary to take the menstrual phase into account when testing females. In future research, we plan to add females to our sample, if they agree to indicate the day/phase of the cycle in the questionnaire.

## Figures and Tables

**Table 1:** The results of psychological testing before the main stage of the study: the Spielberger-Hanin scale of situational and personal anxiety (the State-Trait Anxiety Inventory (STAI) [39]) and the Toronto Alexithymia Scale (adaptation for Russia) [40].

Data	Mean	St. dev.	Median	Min	Max
Alexithymia	37.18	10.58	33	24	62
State anxiety	33.24	8.49	33	21	52
Trait anxiety	30.53	8.44	30	20	52

## Data Availability

Data are available by contacting the corresponding author.
